# Anxiolytic-like Effect of Testosterone in Male Rats: GABA_C_ Receptors Are Not Involved

**Published:** 2011

**Authors:** Ali Roohbakhsh, Akbar Hajizadeh Moghaddam, Karim Mahmoodi Delfan

**Affiliations:** 1 Physiology and Pharmacology Research Center, Rafsanjan University of Medical Sciences, Rafsanjan, Iran; 2 Department of Biology, Faculty of Basic Sciences, University of Mazandaran, Babolsar, Iran; 3 Department of Biology, Tarbiat Moallem University, Tehran, Iran

**Keywords:** Anti-Anxiety Agents, GABA-C receptors, Testosterone, Infusions, Intraventricular

## Abstract

**Objective(s):**

The effect of testosterone on anxiety-like behaviors has been the subject of some studies. There is evidence that testosterone modulates anxiety via GABA (gama aminobutyric acid) and GABAergic system. The involvement of GABA_C_ receptors in those effects of testosterone on anxiety-like behaviors of the rats was investigated in the present study.

**Materials and Methods:**

A group of rats received subcutaneous injections of testosterone (5, 10 and 20 mg/kg). Two groups of rats received intracerebroventricular injections of either CACA (GABA_C_ agonist, 0.125 μg/rat) or TPMPA (GABA_C_ antagonist, 3 microg/rat) following administration of testosterone (5, 10 and 20 mg/kg). After the injections, the rats were submitted to the elevated plus-maze test of anxiety.

**Results:**

The rats received testosterone alone, showed a decreased in anxiety-like behaviors (*P*< 0.01). Administration of either CACA or TPMPA did not modify animals’ behavior compared to the rats received testosterone alone.

**Conclusion:**

The results of the present study showed that administration of testosterone induces anxiolytic-like behaviors in the rats and GABA_C_ receptors possibly are not involved in the anxiolytic effect of testosterone.

## Introduction

There are both basic and clinical reports showing that steroid hormones are involved in the modulation of anxiety. Indeed, women are 85% more likely to experience anxiety disorders than men are (1), suggesting that androgens may protect men against anxiety disorders. A wide range of studies have implicated androgens in the modulation of anxiety disorders. In animal studies, the anxiolytic-like actions of androgens either following acute (2-4) or long chronic (3-6) administration have been observed. Conversely, the anxiogenic effects of anabolic-androgenic steroids have also been documented (7,8). Testosterone interacts with intracellular androgen receptors (9), suggesting that its anxiolytic activity could be mediated by this mechanism. Meanwhile, there is increasing evidence that certain steroids may alter neuronal excitability via interaction with cell membrane receptors (10). For example, it has been demonstrated that the reduced testosterone metabolites, androstanediol and androsterone, have little or no affinity for androgen receptors (11), but they are potent GABA_A_ (gama aminobutyric acid A) receptor agonists and have GABA-mediated functions (5). This finding is suggesting that testosterone possibly exerts its behavioral actions via conversion to these reduced metabolites and this may explains the mechanism by which GABA_A_ receptor antagonists, picrotoxin and bicucculine, block the anxiolytic effect of testosterone (2). Therefore, GABAergic system may be a candidate for the mediation of many of the behavioral effects of anabolic androgenic steroids.

Gama aminobutyric acid is the main inhibitory neurotransmitter in the mammalian central nervous system. It exerts its effects through three distinct classes of membrane receptors: GABA_A_, GABA_B_ and GABA_C_ (12). GABA_C_ receptors appear to be much simpler than GABA_A_ receptors and are more sensitive to GABA than GABA_A_ receptors, but they have not been studied as extensively as GABA_A_ receptors (13). They have been implicated in visual processing, regulation of sleep-waking rhythms, pain perception, memory, learning, regulation of hormones and neuroendocrine gastrointestinal secretion (14). Beside the GABA_A_ receptors, GABA_B_ receptors have also been considered in the modulation of some neuroendocrine effects of testosterone (15). The interaction of GABA_C_ receptors with testosterone and its metabolites has not been studied well. There is little evidence that shows such interaction may exist. For example, along with GABA_A_ and GABA_B_ receptors, high density of GABA_C_ receptors exists in male reproductive tissues and GABA_C_ receptors facilitate rat sperm acrosome reaction (14). Moreover, testosterone can be aromatized to estradiol which has been reported as potent inhibitor of the GABA_C_ receptors (16). On the basis of the above evidence, we evaluated the effect of testosterone on anxiety-like behaviors of the rats and the possible involvement of GABA_C_ receptors in those effects of testosterone on anxiety-like behaviors.

## Materials and Methods

***Animals***

Male Wistar rats from Pasteur institute, , weighing 200-250 g were used in the present study. Animals were housed 5 per cage, in a room with a 12:12 hr light/dark cycle (lights on 07:00 hr) and controlled temperature (23±2 ^o^C). Animals had access to food and water* ad libitum*. Each experimental group included eight animals. All experimental procedures were carried out according to a protocol approved by the local Animal Ethics Committee.

***Surgery***

Rats were anesthetized intraperitoneally with ketamine hydrochloride (50 mg/kg) and xylazine (4 mg/kg) and fixed in a stereotaxic frame. The stainless steel guide cannula (22-gauge) was implanted unilaterally in the right lateral cerebral ventricle region (coordinates: Anterior-posterior: -0.8 mm; Medial-lateral: +1.6 mm; Ventral: -3.5 mm) according to Paxinos and Watson (17). It was then fixed to the skull with acrylic dental cement. 

***Drugs***

The drugs used in the present study were testosterone propionate [Iran Hormone, , ], CACA (selective GABA_C_ receptor agonist) and TPMPA (selective GABA_C_ receptor antagonist) [Tocris, ]. Testosterone was dissolved in sesame oil. CACA and TPMPA were dissolved in sterile 0.9% saline. 

***Procedure***

Intracerebroventricular (i.c.v.) injections were performed by means of an internal cannula (27-gauge, Supa; ), terminating 1.5 mm below the tip of the guide cannula, connected by polyethylene tubing to a 2-µl syringe. Rats were hand-held as the experimenter inserted the injector. On each side, 2 µl solution was infused over a 60 sec period. To allow diffusion of the solution and to reduce the possibility of reflux, the injector was left in place for an additional 60 sec. 

***Elevated plus-maze test of anxiety***

The elevated plus-maze (EPM) comprised 2 open arms (50×10 cm) and 2 enclosed arms (50×10×40 cm) extended from a common central platform (10×10 cm). The apparatus, constructed from wood, was elevated 50 cm above floor level. Testing was conducted in a quiet room between 9.00 a.m. and 13.00. At 5 days following surgery, rats were brought into the behavioral testing room and left undisturbed for at least 1 hr prior to testing. The rats were individually placed in the center of the maze facing an open arm and allowed 5 min of free exploration. After each test, the floor was cleaned with distillated water. Measures were the frequencies of total, open, and closed arm entries (arm entry= all 4 paws into an arm) and the time spent in open, closed, and central parts of the maze. The percentage of open arm entries (%OAE) and open arm time (%OAT) as the standard indices of anxiety-like behaviors were calculated (18). A significant decrease in the percentage of time in open arms and/or open arm entries was indicative of an increased level of anxiety. Total arm entries were measured as a relative pure index of locomotor activity (18).

***Experiments***

*Experiment 1: The effect of testosterone on anxiety-like behaviors*

**Figure 1.**
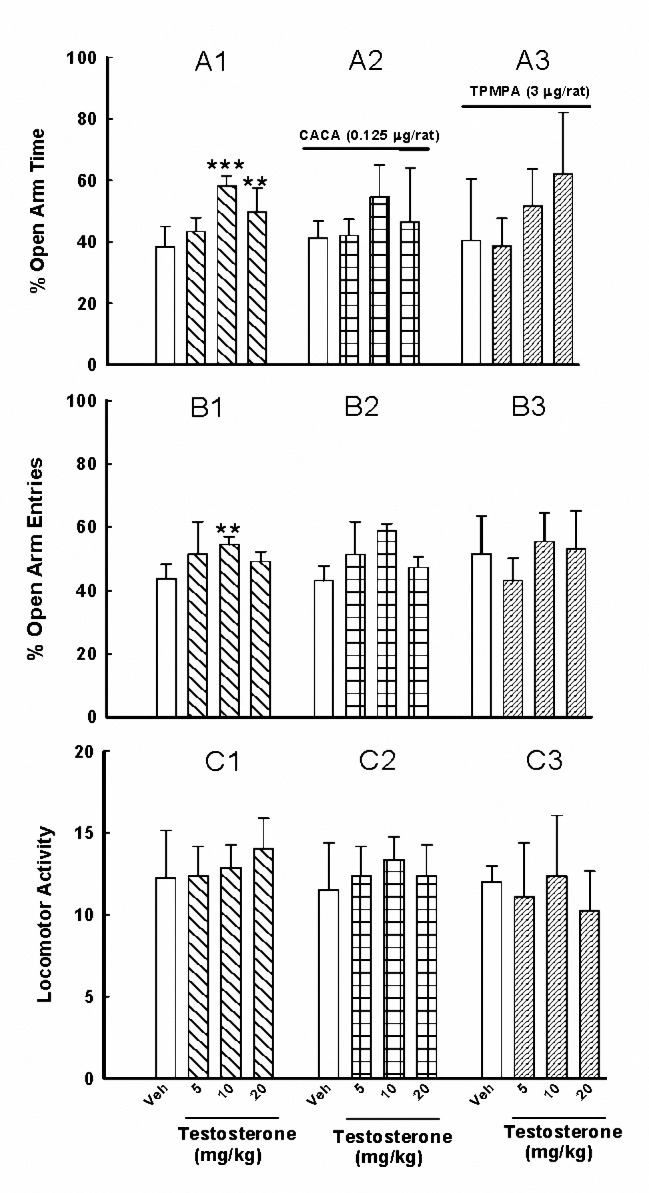
Effects of subcutaneous injections of testosterone alone or in the presence of CACA or TPMPA on anxiety-like behaviors. Rats received testosterone (5, 10 and 20 mg/kg, Figures A1, B1, C1) or intracerebroventricular injections of either CACA (0.125 µg/rat, Figures A2, B2, C2) or TPMPA (3 µg/rat, Figures A3, B3, C3) after subcutaneous injections of testosterone (5, 10 and 20 mg/kg). Each bar is mean±SD, n= 8. **: *P*< 0.01 and ***: *P*< 0.001 when compared to the vehicle treated rats (1 ml/kg).

The animals received subcutaneous injections of vehicle (1 ml/kg) or one of the three doses of testosterone (5, 10 and 20 mg/kg). The test session was performed 24 hr after subcutaneous injections. %OAT, %OAE and locomotor activity were measured (Figure 1, A1, B1, C1).

*Experiment 2: The effects of GABAc receptor agonist and antagonist on the anxiolytic-like effect of testosterone*

Two groups of rats received subcutaneous injections of vehicle (1 ml/kg) or one of the three doses of testosterone (5, 10 and 20 mg/kg). After 24 hr and 5 min before the test, all rats received i.c.v. injections of either CACA (0.125 µg/rat) or TPMPA (3 µg/rat). After the second injections, the animals were exposed to the EPM test. (Figure 1, A2, B2, C2 & A3, B3, C3).

***Verification of cannula placements***

After completion of the experimental sessions, rats received 2 µl of methylene blue. Approximately 10 min after the injection, the animals were decapitated and their brains were removed, blocked and cut coronally through cannula placements. Data from the rats with injection sites located outside the ventricle were not used in the analysis.

***Statistical analysis***

One-way ANOVA was used for the comparison between the effects of different doses of drugs with vehicles. Two-way ANOVA was used for evaluation of interactions between drugs. Following a significant *F*-value, *post-hoc *analysis (Tukey-test) was performed for assessing specific group comparisons. Differences with *P*< 0.05 between experimental groups at each point were considered statistically significant.

## Results

In the first experiment, testosterone at the doses of 10 and 20 mg/kg increased %OAT [Figure 1, A1; *F*_(3,28)_ = 17.4, *P*< 0.001]. It also increased %OAE at the dose of 10 mg/kg [Figure 1, B1; *F*_(3,28)_= 4.63, *P*< 0.01]. Testosterone produced no significant change in the locomotor activity [Figure 1, C1; *F*_(3,28)_ = 1.12, *P*> 0.05]. This finding is suggesting an anxiolytic-like effect for testosterone.

In the second experiment (Figure 1, A2, B2, C2), the effects of CACA with testosterone on %OAT [*F*_(3,56)_= 0.48, *P*>0.05] and %OAE [*F*_(3,56)_= 0.28, *P*> 0.05] were not significant when compared with the animals received testosterone alone. Moreover, i.c.v. injection of TPMPA with testosterone did not affect animal behavior when compared with the animals received testosterone alone; %OAT [*F*_(3,56)_= 0.7, *P*> 0.05] and %OAE [*F*_(3,56)_= 2.5, *P*> 0.05]; (Figure 1, A3,B3,C3). These findings are suggesting that GABA_C_ receptors possibly are not involved in those effects of testosterone on anxiety-like indices.

## Discussion

In the present study, administration of testosterone produced a clear anxiolytic-like action in the elevated plus-maze test. This finding is consistent with previous reports showing that a single systemic exposure to androgens induces anxiolytic-like effects in rodents (2, 3,19). Decrease in anxiety-like behaviors and enhancement of the cognitive performance of aged male mice have also been reported following administration of androgens (20). In another study, intra-dorsomedial hypothalamus infusion of 17α-methyltestosterone has shown anxiolytic-like behaviors in female rats (21). On the other hand, Edinger and Frye (22) showed that intrahippocampal infusion of flutamide, a testosterone receptor antagonist, produced anxiogenic-like behaviors in male rats. Meanwhile, female mice and rats spend less time on the open arms of the elevated plus-maze than do male mice and rats representing more anxious behaviors (23,24). In line with these reports, an increase in vulnerability of women to anxiety disorders has been linked to lower level of endogenous testosterone compared to men. Indeed, the rise in illicit androgen use may also be related to androgens’ anti-anxiety effects (25). Furthermore, it has been reported that in male rodents, removal of the primary source of endogenous androgens through gonadectomy increases anxiety-like behaviors in the open field and elevated plus-maze (4). In the same way, young hypogonadal men, with low endogenous testosterone levels, exhibit decreased performance in cognitive tasks and are more likely to be diagnosed with an anxiety or depressive disorder (26). In contrast, some studies reported that administration of androgens is anxiogenic (7,8) or has no effect on anxiety-related behaviors in rodents (27). These inconsistent and contradictory results may be due to variation in species, sex and age of the subjects, as well as in environmental vhariables and the types and regimes of administered androgens.

Despite well-documented studies of the influence of testosterone on behavior, the mechanisms underlying the behavioral effects of this hormone and other steroid hormones need to be explored. Therefore, the GABAergic system has been proposed as an attractive candidate for mediating many of these effects. In 2002, Aikey *et al* (2) found that picrotoxin and bicuculline, as GABA_A_ receptor antagonists, blocked the anxiolyic-like effect of testosterone. In a different study, Reddy and Jian (28) showed that androstanediol, a testosterone-derived metabolite, is an activator of GABA_A_ receptors. By the way, there is evidence that anabolic androgenic steroids may also affect GABAergic system via GABA_C _receptors (see introduction). To test this hypothesis, we evaluated the effect of intracerebroventricular injections of a selective GABA_C_ receptor agonist (CACA) and a selective GABA_C_ receptor antagonist (TPMPA) on the anxiolytic action of the testosterone. Intracerebroventricular administrations were done because there is evidence that these drugs can not cross blood brain barrier (29). Recently, we reported that i.c.v. administration of CACA and TPMPA alone produced significant anxiogenic and anxiolytic-like effects in male rats respectively (30). The doses of CACA and TPMPA that we used in the present study are ineffective doses of these drugs on anxiety-like behaviors. However, to our knowledge this study is the first attempt to find an interaction between testosterone and GABA_C_ receptors in an *in vivo* experiment, but the results of the present study failed to show such interaction. This means that GABA_C_ receptors possibly are not mediating the anxiolytic effect of testosterone. GABA_C_ receptors are a class of GABA receptors that have not been studied well. Especially, their possible role(s) in the brain has not evaluated well so far. However, their role in the regulation of sleep (31), memory (32) and anxiety (30) has been reported. The results of a recent study showed that estradiols, the product of aromatization of testosterone, are effective inhibitors of the GABA_C_ receptors (16). This is suggestive that other steroids may affect GABAergic transmission in the nervous system via GABA_C_ receptors. This hypothesis needs to be explored in an *in vivo* study. 

## Conclusion

The results of this study showed that testosterone produced a significant anxiolytic-like effect and GABA_C_ receptors possibly are not mediating this effect of testosterone. 
